# LRIG1 as a Potential Novel Marker for Neoplastic Transformation in Ocular Surface Squamous Neoplasia

**DOI:** 10.1371/journal.pone.0093164

**Published:** 2014-04-07

**Authors:** Maho Nagata, Takahiro Nakamura, Chie Sotozono, Tsutomu Inatomi, Norihiko Yokoi, Shigeru Kinoshita

**Affiliations:** 1 Department of Ophthalmology, Kyoto Prefectural University of Medicine, Kyoto, Japan; 2 Research Center for Inflammation and Regenerative Medicine, Doshisha University, Kyoto, Japan; Ohio State University Medical Center, United States of America

## Abstract

The leucine rich repeats and immunoglobulin-like protein 1 (LRIG1) is a newly discovered negative regulator of epidermal growth factor receptor (EGFR) and a proposed tumor suppressor. It is not universally downregulated in human cancers, and its role in neoplastic transformation and tumorigenesis is not well-documented. In this study, we show the expression of LRIG1 as a novel potential marker for neoplastic transformation in ocular-surface squamous neoplasia (OSSN). The following two groups were included in this study: 1) benign group (3 cases; 1 with papilloma and 2 with dysplasia) and 2) malignant group (3 cases with squamous cell carcinoma (SCC)). In both groups, immunofluorescence analysis was firstly performed for keratins 4, 12, 13, and 15 to characterize the state of differentiation, and for Ki67 to evaluate the proliferation activity. Subsequently, LRIG1 and EGFR expression was analyzed. Either keratin 4 and/or 13, both non-keratinized epithelial cell markers, were generally expressed in both groups, except for 1 severe SCC case. Keratin 15, an undifferentiated basal cell marker, was more strongly expressed in the malignant cases than in the benign cases. The Ki67 index was significantly higher (*P*<0.002) in the malignant group (33.2%) than in the benign group (10.9%). LRIG1 expression was limited to basal epithelial cells in normal corneal epithelial tissue. Interestingly, LRIG1 was expressed throughout the epithelium in all the benign cases. In contrast, its expression was limited or totally disappeared in the malignant cases. Inversely, EGFR staining was faintly expressed in the benign cases, yet strongly expressed in the malignant cases. Malignant tissue with proliferative potential presented EGFR overexpression and inverse downregulation of LRIG1, consistent with LRIG1 being a suppressor of neoplastic transformation by counteracting the tumor growth property of EGFR. Our findings indicate that downregulation of LRIG1 is possibly a novel potential marker of transformation and tumorigenesis in OSSN cases.

## Introduction

Ocular surface squamous neoplasia (OSSN) is a dysplastic or carcinomatous lesion arising from epithelial cells in the conjunctiva, limbus, or cornea, and histologically encompasses a spectrum from simple dysplasia to carcinoma in situ (CIS) to invasive squamous cell carcinoma (SCC) [1]. The incidence of OSSN ranges from 0.13 to 1.9 cases per 100,000 people per year [2], [3]. Simple dysplasia and CIS occurs as a localized lesion confined to the surface epithelium, whereas SCC occurs as a more invasive lesion that has broken through the basement membrane and has invaded the underlying stroma [4]. Uncommonly, lesions that are untreated or incompletely excised can reportedly develop intraocular invasion [5] and metastasis [6]. Symptoms can range from none at all to severe pain and visual loss [1], and OSSN patients most commonly present with red eye and ocular irritation [7]. The lesions are often diagnosed as hyperplasia, pterygium, leukoplakia, pinguecula, papilloma, granuloma, or a type of tumor [8]. Dysplasia, CIS, and invasive SCC are clinically similar in appearance, so it is difficult to distinguish between them in the clinical setting [4]. Therefore, their pathological grade must be judged via histopathological examinations. To the best of our knowledge, there is no previous report of a neoplastic transformation marker that elucidates a distinct difference between benign and malignant tissue in OSSN.

Interestingly, it has been reported that OSSN lesions commonly occur in the limbus of the interpalpebral fissure [8], [9]. Limbal stem cells (LSCs) are harbored in the limbus [10] and are thought to be slow cycling and undergo infrequent division in vivo, however, they have the potential for self-renewal and rapid proliferation and differentiation under the proper conditions, such as in wound healing or in tissue culture [11], [12]. Stem cells have often been considered to be associated with cancer cells. Although it is impossible to ascertain with certainty the origin of cancer cells, some studies have reported that malignant tumors originate from the transformation of tissue stem cells via mutations that lead to dysregulation of the normal mechanisms that control stem-cell growth and proliferation [13], [14]. The limbus is the only stem-cell-rich area of the human body that is easy to observe, and is an area in which cancer can be detected even at the very first stage of tumor growth. Thus, studies of OSSNs are thought to have crucial importance in tumorigenesis research [15].

In our recent study, we found that leucine rich repeats and immunoglobulin-like protein 1 (LRIG1) was highly expressed in holoclone-type corneal epithelial stem cells, and that it was essential for the cell-fate maintenance of corneal epithelium during tissue repair [16]. Moreover, examination of the expression pattern of LRIG1 showed us that it is sporadically expressed in the basal cells of ocular surface epithelium, indicating its relationship with epithelial stem/progenitor cell-related molecules. LRIG1 is reportedly a negative regulator of epidermal growth factor receptor (EGFR) signaling [17], [18], and it has recently been identified as a marker of human epidermal [19] and intestinal [20], [21] stem cells. In 2006, Jensen et al. found that LRIG1 expression is associated with patches of basal and hair follicle cells, corresponding to the foci of epidermal stem cells, suggesting that LRIG1 regulates epidermal stem cell quiescence by downregulating EGFR and suppressing stem cell proliferation [19]. In addition, Powell et al. and Wong et al. reported in 2012 that LRIG1 marks intestinal stem cell populations and serves a functional role in the maintenance of small intestinal and colonic epithelial homeostasis [20], although Powell's group showed that these cells are predominantly quiescent, whereas Wong's group reported that the majority of LRIG1-positive intestinal stem cells are proliferating cells [21].

The human LRIG1 gene is located at chromosome 3p14.3, which is a region frequently deleted in human cancers, suggesting that LRIG1 might be a tumor suppressor gene [22], [23]. In fact, the findings of previous reports have shown that LRIG1 is downregulated in several types of tumors, such as cutaneous squamous cell carcinoma [24], renal cell carcinoma [25], glioblastoma [26], and breast cancer [27]. To date, the tumor suppressing function of LRIG1 has mainly been attributed to its inhibitory effect on EGFR signaling [25]–[29]. Although the precise mechanism of LRIG1 downregulation in cancer cells remains unclear, the findings of a recent study demonstrated that LRIG1 forms a ternary complex between LRIG1, E-cadherin, and EGFR, which upon cell-to-cell contact negatively regulates EGFR signaling [30]. Recent studies have shown that LRIG1 function has a therapeutic potential for cancer and is also strongly related to EGFR signaling, as ectopic expression of LRIG1 reportedly inhibits oncogenic EGFR mutant EGFRvIII expression in glioblastoma [26] and the downstream MAPK and AKT signaling pathway in glioma [29]. Soluble ectodomains of LRIG1 are reportedly sufficient to inhibit the growth of cancer cells by attenuating EGFR activity [27], and more recently, a study showed that the LRIG1 ectodomain can be naturally shed and can function as a non-cell-autonomous regulator of EGFR signaling [31]. In view of these findings, the function of LRIG1 and EGFR are considered to be closely linked with one another.

In this present study, the possible function of LRIG1 on tumor progression was investigated via immunofluorescence analysis in human OSSN. Immunofluorescence analysis clearly revealed the downregulation of LRIG1 in malignant tissues, and inversely, the upregulation of LRIG1 in benign tissues. Thus, and to the best of our knowledge, the findings of this study show for the first time that LRIG1 is a potential novel neoplastic transformation marker that distinguishes benign from malignant tumors in human OSSN.

## Materials and Methods

All experimental procedures performed in this study were approved by the Institutional Review Board for Human Studies of Kyoto Prefectural University of Medicine, Kyoto, Japan. Prior verbal and written informed consent was obtained from each patient in accordance with the tenets set forth in the Declaration of Helsinki for research involving human subjects.

### Subjects

Six patients with OSSN and papilloma who underwent excision and tissue biopsy at Kyoto Prefectural University of Medicine were enrolled in this study. The clinical diagnoses were made by a cornea specialist and were histologically confirmed by a pathology report. Histological diagnosis was based on identification of epithelial disarray with disturbances in maturation, alterations in the nuclear to cytoplasmic ratio, and evidence of atypia. Cases were classified as dysplasia if only a partial thickness of the epithelium was replaced or penetrated by abnormal cells. Lesions with dysplastic cells involving the full thickness of the epithelium, including the surface layer, but not penetrating the substantia propria of the Bowman's layer, were classified as CIS. Lesions with abnormal epithelial cells that had invaded the basement membrane and had grown in sheets or cords into the stromal tissue were classified as invasive SCC. As controls, normal conjunctival, limbal, and corneal tissue obtained from human corneoscleral rims from the Northwest Lion Eye Bank (Seattle, WA, USA) were also examined.

### Immunofluorescence

Immunofluorescence analysis of all primary antibodies was performed via the indirect immunofluorescent technique. Sectioned samples were immediately washed with phosphate-buffered saline (PBS) and embedded in optimal cutting temperature (OCT) compound. Frozen sections were sliced to the thickness of 8 µm, placed on gelatin-coated slides, air dried, and fixed in 100% acetone or 4% paraformaldehyde at 4°C. After several washings with PBS, the sections were incubated with 1% bovine serum albumin at room temperature (RT) for 30 minutes to block nonspecific binding. They were then incubated with the appropriate primary antibodies for 1 hour at RT, and washed 3 times in PBS containing 0.15% TRITON X-100 (The Dow Chemical Company, Midland, MI, USA) for 15 minutes per wash. For negative control experiments, the equivalent serum was used. After staining with the primary antibodies, the sections were incubated at RT for 1 hour with appropriate fluorescein-conjugated secondary antibodies. After several washes with PBS, they were coverslipped with mounting medium containing propidium iodide (PI; Vector Laboratories, Inc., Burlingame, CA, USA). The slides were then examined by confocal microscopy (FluoView; Olympus Corporation, Tokyo, Japan).

### Antibodies

The following antibodies were used for the immunostaining analysis: mouse monoclonal anti-cytokeratin type 4 and 13 antibodies (Novocastra Laboratories Ltd., Newcastle upon Tyne, UK), rabbit polyclonal anti-cytokeratin 12 (TransGenic Inc., Kumamoto, Japan), mouse monoclonal anti-cytokeratin 15 (Abcam plc, Cambridge, UK), mouse monoclonal anti-Ki67 antibody (BD Biosciences, San Jose, CA, USA), rabbit polyclonal anti-LRIG1 antibody (courtesy of Dr. Satoshi Itami, Osaka University, Osaka, Japan) [16], [19], [32], and goat anti-EGFR antibody (Sigma-Aldrich CO. LLC., St. Louis, MO, USA).

### Ki67 Labeling Index

Since Ki67 protein is reportedly present during the G1, S, G2, and M phases of the cell cycle and is strictly associated with cell proliferation [33], the number of Ki-67-positive cells in the tumor cells was counted. First, the sections were immunohistochemically stained with the above-mentioned Ki67. The areas for cell counting were then selected from the most mitotically active parts of the tumors. Three representative images were taken from each patient. Next, the cells positive for Ki67 and PI were manually counted and the percentage of Ki67-positive cells amongst the total cell count was calculated.

### Statistics

The Student's unpaired t-test was used to evaluate the statistical differences between the groups, and *P*-values of <0.05 were considered statistically significant.

## Results

### Clinical Presentation

**Figure 1:**
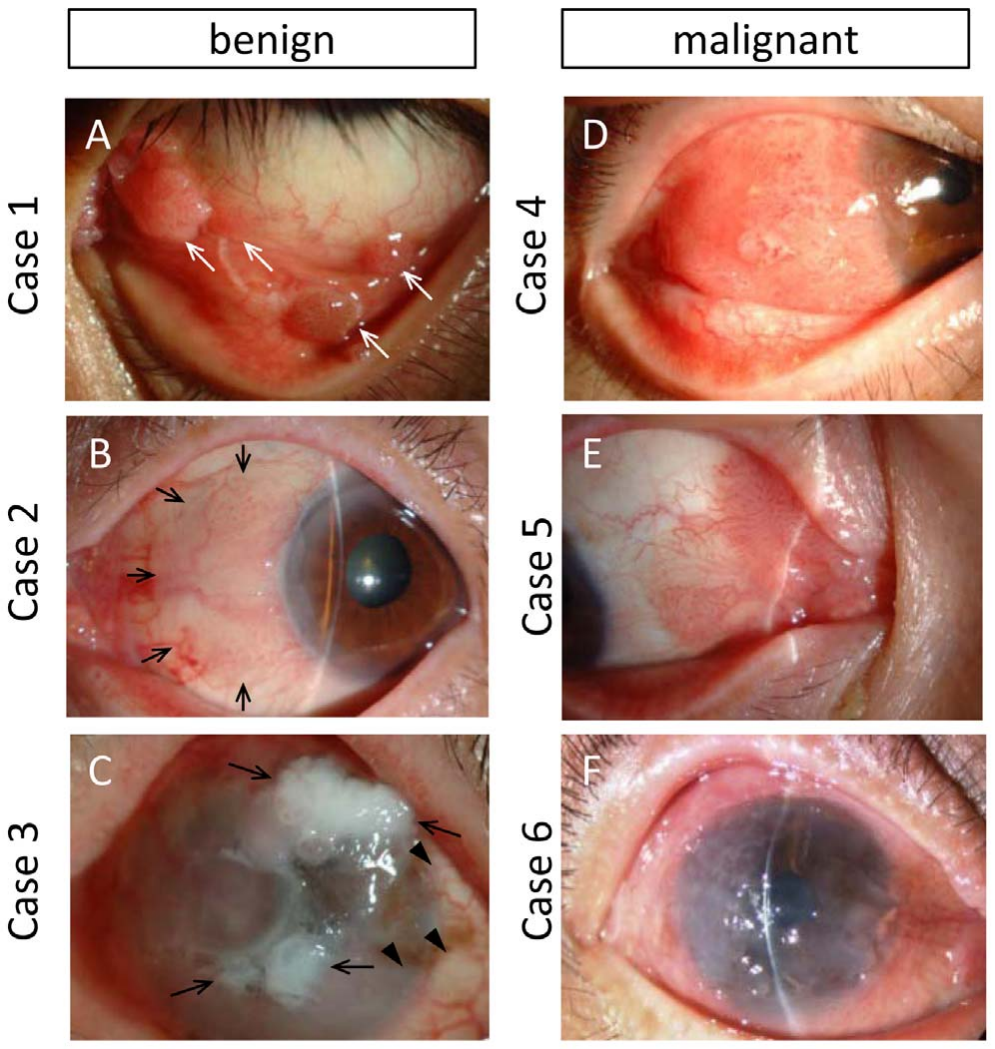
Clinical presentation of 6 cases with OSSN. (A) Case 1 with papilloma. Multiple sessile masses were prominent in lower conjunctival fornix and palpebral conjunctiva (arrows). (B) Case 2 with severe dysplasia. Elevated smooth mass lesion in nasal side of limbus to bulbar conjunctiva (arrows) presented gelatinous surface with typical corkscrew vascular tufts. (C) Case 3 with severe dysplasia. Irregularly elevated mass was diffusely covered whole ocular surface. Flat gelatinous lesion and irregular whitish lesion (arrows) were mixed. Lightly pigmented area was also observed (arrowheads). (D) Case 4 with CIS. Elevated nodular mass with vascular tufting was covered temporal side of limbus to bulbar conjunctiva. (E) Case 5 with CIS. Nodular lesion with typical vascular tufts extended from the caruncle to bulbar conjunctiva. (F) Case 6 with invasive SCC. Gelatinous irregular epithelium covered whole ocular surface.

The clinical data of the 6 cases (4 males and 2 females) are listed in Table 1. The age distribution of the patients at the time of operation ranged from 36 to 92 years (mean age: 63.8 years). In all 3 of the benign OSSN cases the lesion occurred in the left eye, and in all 3 of the malignant OSSN cases the lesion occurred in the right eye. The lesion was located at the nasal side in 2 cases (cases 2 and 5), at the temporal side in 1 case (case 4), and at the lower fornix in the 1 papilloma case (case 1). Nearly the entire ocular surface was diffusely covered by the tumor in 2 cases (cases 3 and 6), and the limbal area was affected in 4 cases (cases 2–5). The clinical appearance was gelatinous in 5 cases (cases 2–6) and was papilliform in the 1 papilloma case (case 1) (Fig. 1). Case 3 presented with a partial leukoplakic lesion (Fig. 1C). The lesion was nodular in 4 cases (cases 1, 2, 4, and 5), yet diffuse in 2 cases (cases 3 and 6). Characteristic tufted superficial vessels were observed in 3 nodular cases (cases 2, 4, and 5). The elevated mass(es) were totally dissimilar from the normally differentiated ocular surface epithelial cells in both groups, however, it was difficult to distinguish between the groups based on the clinical appearance alone.

**Table 1:** Patient Demographics and Clinicopathological Features.

Patient No.	Age (yrs)	Gender	Affected Eye	Lesion Site	Clinical Appearance	Histopathological Diagnosis	Group
1	36	Male	Left	Lower fornix, multiple	Nodular, papilliform	Papilloma	Benign
2	76	Female	Left	Nasal limbus to bulbar conjunctiva	Nodular, gelatinous	Severe dysplasia	Benign
3	92	Male	Left	Diffuse	Diffuse, gelatinous (partially leukoplakic)	Severe dysplasia	Benign
4	60	Male	Right	Temporal limbus to bulbar conjunctiva	Nodular, gelatinous	CIS	Malignant
5	61	Male	Right	Caruncle to nasal bulbar conjunctiva	Nodular, gelatinous	CIS	Malignant
6	58	Female	Right	Diffuse	Diffuse, gelatinous	Invasive SCC	Malignant

CIS = carcinoma in situ; SCC = squamous cell carcinoma.

### Pathology

**Figure 2:**
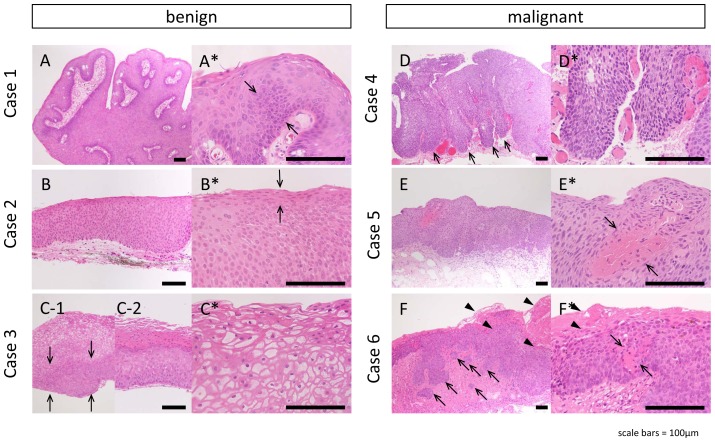
Histopathology of 6 OSSN cases. Histopathology of 6 cases with lower magnification (A–F) and higher magnification (A′–F′). (A) Case 1. Thick, acanthotic squamous epithelium with fibrovascular cores. Some cells had more hyperchromatic nuclei, suggesting a mild degree of dysplasia (arrows in A′). (B) Case 2. Almost all the layers were replaced by large squamous cells, but surface layer presented differentiation (arrow in B′). (C) Case 3. The bottom third to half of the epithelium was transformed to small epithelial cells (arrows). Koilocytes were identified in upper layer (C′). Unlike benign cases, the full thickness of the epithelium was replaced by atypical cells in case 4–6 (D–F). (D) Case 4. Multiple epithelial lobules extended into the subepithelial connective tissue (arrows). Cells have atypical appearance, with darker cytoplasm and pleomorphic nuclei (D′). (E) Case 5. The basement side was irregular with increased inflammatory responses in underlying connective tissue. Within the epithelium, there was abnormal keratinization with central foci of acellular keratin (arrows in E′). (F) Case 6. The infiltrating lobules, nests, and cords were extending from the basement line into the underlying connective tissue with lymphocytic infiltrate within the connective tissue (arrows). The surface hyperkeratosis was prominent (arrowheads) simultaneously with intraepithelial hyperkeratosis (arrows in F′). Cell atypia is prominent (F′). Scale bars = 100 µm.

Microscopy photographs are shown in Figure 2. The papilloma case (case 1) presented the typical papillary configuration with fibrovascular cores covered by thick hyperplastic epithelium. Some cells were found to have larger and more hyperchromatic nuclei, thus suggesting a mild degree of dysplasia (Fig. 2A and A*). In one of the severe dysplasia cases (case 2), transformation extended toward the surface layers of the epithelium, except for the outer surface layer that is composed of well-differentiated cells (Fig. 2B and B*). In another severe dysplasia case (case 3), the bottom third to one-half of the epithelium was transformed into small epithelial cells (Fig. 2C-1). Koilocytes were identified in the upper layer, thus suggesting human papillomavirus infection (Fig. 2C*). In another section from case 3, hyperkeratosis was prominent, thus suggesting that the section was from a leukoplakic lesion (Fig. 2C-2). In those severe dysplasia cases, the epithelial basal membrane appeared smooth and remained intact without any prominent inflammatory changes in the underlying connective tissue (Figs. 2B and C).

On the other hand, in the malignant cases (cases 4–6), the full thickness of the epithelium was replaced by atypical cells with multiple epithelial lobules extending from the surface neoplastic epithelium into the subepithelial connective tissue that makes the basal membrane irregular (Fig. 2D–F). In 2 cases of CIS (cases 4 and 5), the basal side of the epithelium was comparatively demarcated from the connective tissue (Fig. 2D and E), whereas in 1 case of invasive SCC (case 6), the infiltrating lobules, nests, and cords were found to extend from the basement line into the underlying connective tissue with increased inflammatory responses (Fig. 2F). As is shown in Figure 2E*, the epithelium displayed abnormal keratinization with the cells arranged concentrically around a central focus of acellular keratin. In addition, there was marked hyperkeratosis in case 6 (Fig. 2F*). In these malignant cases, mitotic active cells were found with abnormal mitotic figures haphazardly distributed within the lobules (Fig. 2D*–F*). In view of these findings, the lesions could be clearly distinguished as either benign or malignant only after they were histopathologically examined.

### Immunofluorescence

#### Keratin Expression Pattern

**Figure 3:**
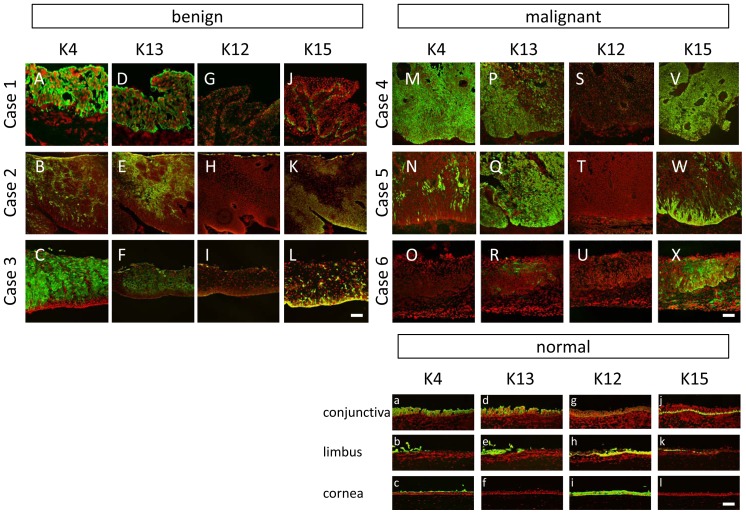
The expression pattern of keratins. The expression pattern of keratins 4 (A–C, M–O), 13 (D–F, P–R, d–f), 12 (G–I, S–U), 15 (J–L, V–X) in the 6 cases; case 1 (A, D, G, J), case 2 (B, E, H, K), case 3 (C, F, I, L), case 4 (M, P, S, V), case 5 (N, Q, T, W), case 6 (O, R, U, X). The expression pattern of these keratins in conjunctivas (a, d, g, j), limbus (b, e, h, k), and corneas (c, f, i, l) in healthy controls are also presented. K4 and K13 were partially positive or totally positive in benign cases (A–F). In the CIS cases (case 4 and 5), strongly positive pattern (M, P, Q) and weak positive pattern (N) were combined. In an invasive SCC case (case 6), K4 and K13 were generally negative (O,R). K12 expression was negative in both benign and malignant cases (G–I, S–U). K15 was overexpressed in all the OSSN tissue (J–L, V–X). The expression pattern was comparatively limited to the basal layer in benign cases (J–L). All three malignant cases showed notably positive staining (V–X). Scale bars = 100 µm.

To define the cell biological characteristics of OSSN tissues, the expression patterns of several cytokeratins related to ocular surface epithelium were examined (Fig. 3). K4 and K13, both mucous membrane markers, were found to be positive in the normal (case 1), benign (cases 2 and 3), and CIS (cases 5 and 6) cases (Fig. 3A–F, M, N, P, Q). In contrast, K4 and K13 were found to be nearly negative in a severe SCC case (case 6) (Fig. 3O, R). K12 expression was negative in both the benign and malignant cases (Fig. 3G–I, S–U). K15, a marker of undifferentiated basal cells, was overexpressed in all of the OSSN cases, and the expression pattern was limited to the basal layer in the benign cases (Fig. 3J–L). On the other hand, all 3 malignant cases showed notably positive staining for K15, suggesting that the epithelium was composed of undifferentiated cells that display the basal phenotype (Fig. 3V–X).

#### Cell Proliferating Activity

**Figure 4:**
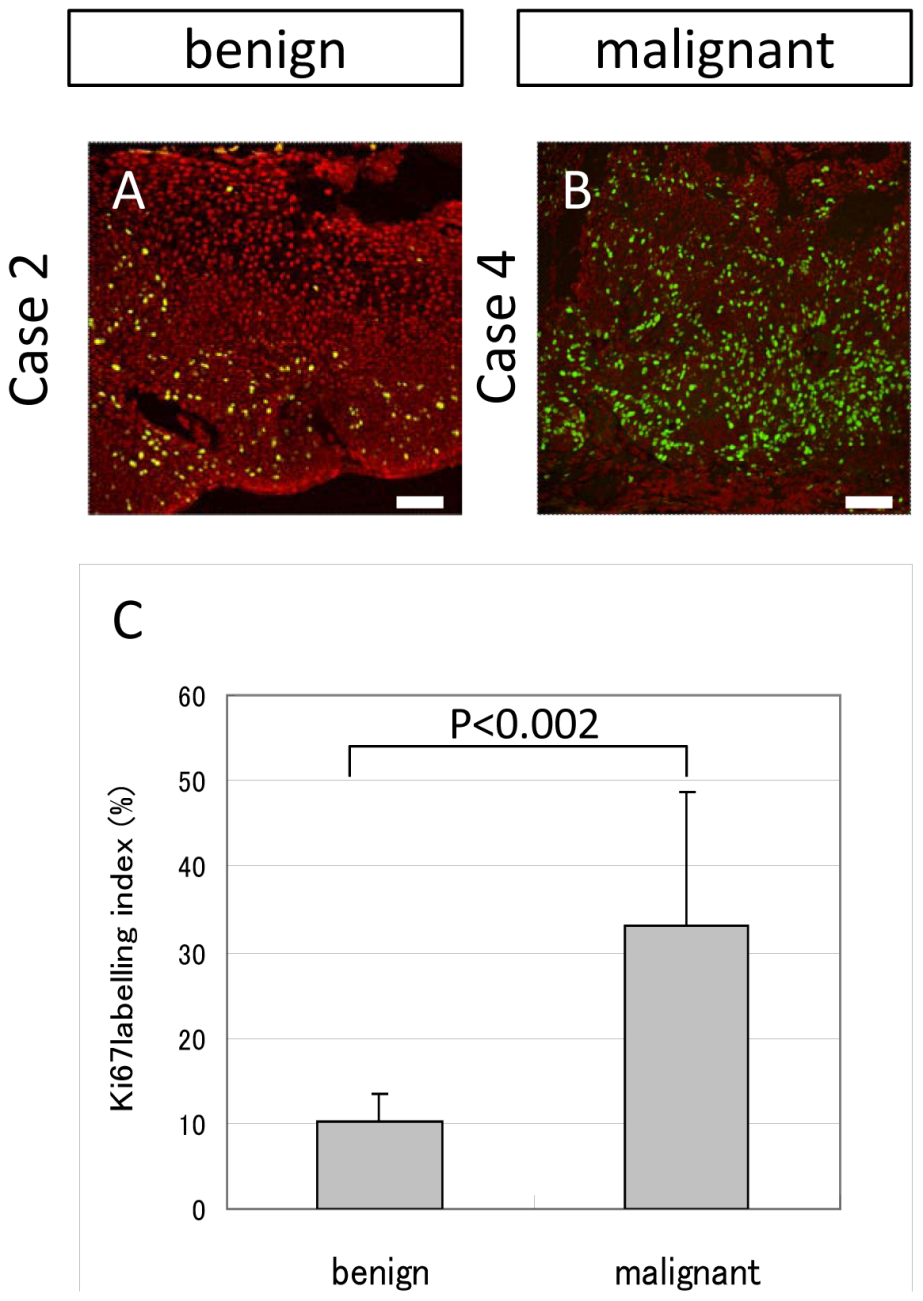
Ki67 expression in OSSN cases. (A and B) Representative expression pattern of benign cases (A, case 2) and malignant cases (B, case 4). Ki67 positive cells were disseminated both in benign and malignant samples. The expression pattern was comparatively sparse and limited in the basal area in the benign samples (A), while diffuse and dense in malignant samples (B). (C) Ki67 labelling index was significantly (p<0.002) high in the malignant tissue (33.2%) rather than benign tissue (10.9%). Scale bars = 100 µm.

Next, Ki67 expression was examined in order to evaluate the proliferative activity of the benign and malignant tumors. Ki67-positive cells were disseminated in both the benign and malignant samples. The expression pattern in the benign samples was comparatively sparse and limited in the basal area (Fig. 4A), however, in the malignant samples the expression was diffuse and dense (Fig. 4B). The Ki67 labeling index (Fig. 4C) was significantly higher (*P*<0.002) in the malignant tissue (33.2%) than in the benign tissue (10.9%). These findings confirm the higher proliferating activity in malignant OSSN cases than in benign OSSN cases.

#### LRIG1/EGFR Expression

**Figure 5:**
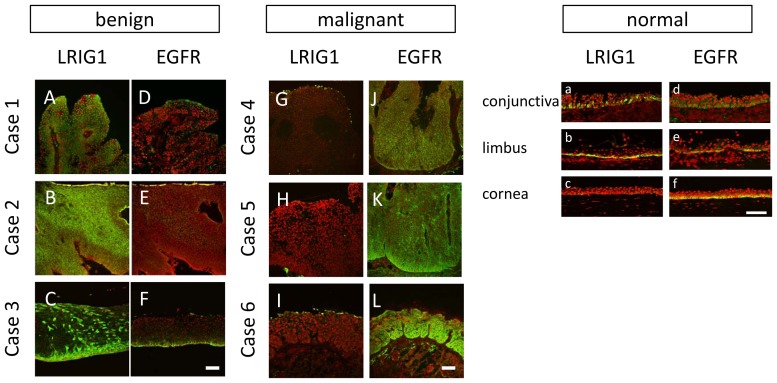
The expression pattern of LRIG1 and EGFR. The expression pattern of LRIG1 (A–C, G–I) and EGFR (D–F, J–L) in case 1 (A, D), case 2 (B, E), case 3 (C, F), case 4 (G, J), case 5 (H, K), and case 6 (I, L). The expression pattern in conjunctivas (a, d), limbus (b, e), and corneas (c, f) in healthy controls are also presented. In benign cases, LRIG1 was overexpressed throughout the layers (A–C). In case 3, koilocytes seemed to be negative for LRIG1 (C). On the other hand, it was generally negative in malignant cases (G–I). Only a small number of LRIG1 positive cells were found in the very surface layer of malignant cases (G–I). On the contrary, EGFR expression was faint in benign cases (D–F), and inversely very strong in malignant cases (J–L). Scale bars = 100 µm.

To confirm the specificity and validity of the LRIG1 antibody, western blot analysis using human recombinant LRIG1 protein was performed (Figure S1). LRIG1 was found to be sporadically expressed in the basal cells of the ocular surface epithelium (Fig. 5a–c). Notably, it was strongly expressed throughout the layers in the benign cases (Fig. 5A–C). In 1 case in the benign group that was clinically severe dysplasia (case 3), koilocytes presented negative for LRIG1, thus making LRIG-positive cells relatively limited to the basal side (Fig. 5C). Surprisingly, there was no LRIG1 expression in the malignant cases (Fig. 5G–I). Moreover, EGFR expression, which is positive in the basal layer of normal ocular surface epithelial cells (Fig. 5d–f), was found to be weak and limited in the basal layer in the benign cases (Fig. 5D–F). On the other hand, EGFR expression was strongly positive throughout the layers in the malignant cases. These findings were opposite to the LRIG1 expression pattern. In view of these findings, a distinctive expression pattern that presents prominent expression in benign tumors (except for koilocytes) and no or only a faint expression in malignant tumors was shown to exist. The expression pattern of EGFR was opposite to that of LRIG1, thus confirming that LRIG1 functions as a negative regulator of EGFR and suggesting that LRIG1 loss produces EGFR overexpression.

## Discussion

The successful treatment of tumors is one of the most important and challenging health-related subjects, and development of therapeutic and diagnostic tools is essential for improving treatment outcomes. Within the limitations of the small sample size, the findings of this study show that LRIG1 expression is downregulated in human conjunctival SCC, and inversely, upregulated in benign tumors, as compared to normal tissue. These novel findings lend support to the suggestion that LRIG1 acts as a tumor suppressor and negative regulator of EGFR [17]–[29]. To date, no previous study has reported such a distinctive expression pattern of LRIG1, indicating that LRIG1 is potentially a novel neoplastic transformation marker that distinguishes benign from malignant tumors in human OSSN.

Our results in malignant OSSN tissue were consistent to those of the previous studies that showed downregulation of LRIG1 and upregulation of EGFR in several types of tumors [25]–[29], [34]. The findings of recent studies revealed that the LRIG1 function regulates the aggressiveness of tumors [35]–[37]. Sheu et al. demonstrated that LRIG1 downregulation leads to malignant phenotypes of head and neck cancer by enhancing EGFR-MAPK-SPHK1 signaling and extracellular matrix remodeling activity [35]. Moreover, Xie et al. reported that the downregulation of LRIG1 promotes the aggressive properties of glioma cells via EGFR/Akt/c-Myc activation [37], and Mao et al. showed that LRIG1 restricts glioma aggressiveness by inhibiting cell proliferation, migration, and invasion [36]. Although these studies did not compare benign and malignant tumors, these findings imply an anti-metastatic role of LRIG1 and thus support our suggestion.

To compare the LRIG1 expression between benign and malignant tumors, Tanemura et al. performed immunohistochemical analysis on well-differentiated and undifferentiated cutaneous SCC and found that LRIG1 expression was decreased in most tumor cells in undifferentiated (high-grade) cutaneous SCC like other tumors, and more interestingly, that it was inversely increased in most cases of well-differentiated (low-grade) cutaneous SCC [24]. These findings closely resemble the findings in this present study. Our findings support the suggestion that the upregulation of LRIG-1 suppresses advances in transformation, whereas complete deletion of LRIG-1 expression induces dedifferentiation of tumor cells. The question why LRIG1 is upregulated in benign or differentiated (low-grade) tumor cannot clearly be answered from the present data, but it is conceivable to theorize that LRIG1 acts within a framework of a negative feedback loop, as previously shown by Gur et al. [17]; i.e., in response to the excessive EGF or EGFR stimulation which makes the cells abnormally proliferate, LRIG1 may overexpress to attenuate the abnormal proliferation to maintain the quiescence of the epithelium.

It should be noted that the pathogenesis of OSSN remains poorly understood. Several risk factors that lead to the development of tumors have been reported, including chronic ultraviolet (UV) exposure [38], [39], the human papilloma virus (HPV) [40], [41], and the human immunodeficiency virus (HIV) [42]. Moreover, it has been reported that multiple factors may contribute to the development of the disease [43]. Among these risk factors, chronic UV exposure is an established cause of disease. Lesions occur exclusively in areas of the eye that are exposed to the sun, and those lesions are associated with solar elastosis and have been shown to contain classical UV-induced p53 mutations [44], [45]. Despite the reliable evidence of UV exposure as an OSSN pathogenesis, the existence of indirect and accumulative mechanisms makes the prevention of the disease difficult. HPV has also been identified as a possible contributing factor [40], [41]. However, benign conjunctival lesions have been shown to contain the infections as well [46], [47]. Moreover, recent studies have demonstrated a zero or low frequency of HPV DNA-positive cases [48], [49]. As for HIV infection, the evidence of a link between HIV seropositivity and OSSN is very strong. However, the precise mechanism of the related tumorigenesis is unclear. The excess risk among HIV-infected individuals suggests the role of another underlying infection. Although an active search for other oncogenic infections is ongoing, no new candidate virus has yet been identified.

Currently, a limited number of molecular studies have been undertaken to reveal the pathogenesis of OSSN. One candidate is the SIRT1 protein, a histone deacetylase, which is reportedly overexpressed in OSSN tissue [50]. In that study, the authors suggested that SIRT1 may play an important role in the genesis and progression of OSSN. More recently, matrix metalloproteinases (MMPs) were found to be overexpressed in SCC tissue, suggesting that MMPs are another candidate OSSN marker [51]. However, since SIRT1 and MMPs are overexpressed even in benign tissue, they can only distinguish pathologic tissue from normal tissue. Clinically, whether the tumor is benign or malignant is of critical importance. Therefore, elucidation of the distinct marker to distinguish malignant tissue from benign tissue is vital.

Expression of EGFR and of their ligands has been demonstrated to occur with high frequency in a majority of human carcinomas, and therefore might play an important role in the pathogenesis of these diseases [52]. EGFR-targeted therapy has been recommended as an efficient strategy for the treatment of several cancers such as non-small cell lung carcinoma [53], colorectal cancer [54], and head-and-neck SCC [55]. Intense expression of EGFR has also been proven in conjunctival SCC, even though the number of patients was small [56]. Therefore, LRIG1 expression as a negative regulator of EGFR plays an important role for finding the mechanism of tumorigenesis or malignant transformation, and for the treatment of patients.

In this present study, and within the limitations of the small sample size, our findings show the downregulation of LRIG1 in malignant OSSN and the inverse upregulation in benign OSSN. In the malignant OSSN cases, EGFR was markedly overexpressed simultaneously with the downregulation of LRIG1, which may cause uncontrolled proliferation and invasion. In benign OSSN cases, the inversely high expression of LRIG1 implies the presence of a negative feedback loop of LRIG1 in response to abnormal proliferation mediated by EGFR. Although the molecular mechanisms underlying the expression pattern have yet to be fully elucidated, LRIG1 could be a key to revealing the mechanism of transformation and tumorigenesis. Further studies are necessary to reveal the role of LRIG1 for the malignant transformation of OSSN.

## Supporting Information

Figure S1 **Western blotting of LRIG1 using rabbit polyclonal anti-LRIG1 antibody.** To confirm the specificity and validity of the LRIG1 antibody, western blot analysis using human recombinant LRIG1 protein was performed.
(TIF)
