# Supplementary figures and images for: LRIG1 as a Potential Novel Marker for Neoplastic Transformation in Ocular Surface Squamous Neoplasia
Source: PLoS One. 2014 Apr 7;9(4):e93164. doi: 10.1371/journal.pone.0093164 (PMC3977825; doi:10.1371/journal.pone.0093164)

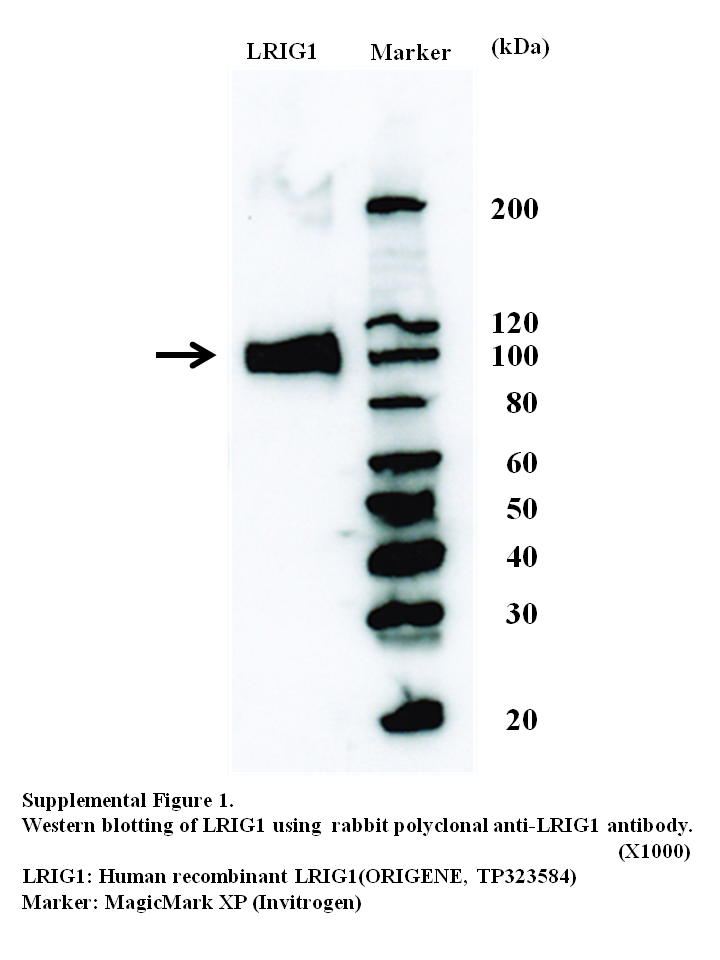

Supplement: Figure S1 — Western blotting of LRIG1 using rabbit polyclonal anti-LRIG1 antibody. To confirm the specificity and validity of the LRIG1 antibody, western blot analysis using human recombinant LRIG1 protein was performed. (TIF) [file pone.0093164.s001.tif]
